# Circulating Tissue Polypeptide-Specific Antigen in Pre-Diagnostic Pancreatic Cancer Samples

**DOI:** 10.3390/cancers13215321

**Published:** 2021-10-23

**Authors:** Emmy Borgmästars, Erik Lundberg, Daniel Öhlund, Hanna Nyström, Oskar Franklin, Christina Lundin, Pär Jonsson, Malin Sund

**Affiliations:** 1 Department of Surgical and Perioperative Sciences/Surgery, Umeå University, 901 85 Umeå, Sweden; erik.lundberg@umu.se (E.L.); hanna.nystrom@umu.se (H.N.); oskar.franklin@umu.se (O.F.); christina.lundin@umu.se (C.L.); malin.sund@umu.se (M.S.); 2 Department of Radiation Sciences/Oncology, Umeå University, 907 37 Umeå, Sweden; daniel.ohlund@umu.se; 3 Wallenberg Centre for Molecular Medicine, Umeå University, 901 87 Umeå, Sweden; 4 Department of Chemistry, Umeå University, 907 36 Umeå, Sweden; par.jonsson@umu.se; 5 Department of Surgery, University of Helsinki, Helsinki University Hospital, 00250 Helsinki, Finland

**Keywords:** TPS, pancreatic ductal adenocarcinoma, circulating biomarkers, early detection, pre-diagnostic cohort

## Abstract

**Simple Summary:**

Detecting cancer early significantly increases the chances of successful (surgical) treatment. Pancreatic cancer is one of the deadliest cancer forms, since it is usually discovered at a late and already spread stage. Finding biomarkers showing pancreatic cancer at an early stage is a possible approach to early detection and improved treatment. The aim of our study was to assess the potential of tissue polypeptide specific antigen (TPS) as a biomarker for early pancreatic cancer detection. We studied TPS levels in blood plasma samples from a population-based biobank in Västerbotten, Sweden that were collected before individuals were diagnosed with pancreatic cancer. Although TPS levels are raised at diagnosis, this occurs late, and thus TPS does not seem to hold promise as an early detection marker for pancreatic cancer.

**Abstract:**

Early detection of pancreatic ductal adenocarcinoma (PDAC) is challenging, and late diagnosis partly explains the low 5-year survival. Novel and sensitive biomarkers are needed to enable early PDAC detection and improve patient outcomes. Tissue polypeptide specific antigen (TPS) has been studied as a biomarker in PDAC diagnostics, and it has previously been shown to reflect clinical status better than the ‘golden standard’ biomarker carbohydrate antigen 19-9 (CA 19-9) that is most widely used in the clinical setting. In this cross-sectional case-control study using pre-diagnostic plasma samples, we aim to evaluate the potential of TPS as a biomarker for early PDAC detection. Furthermore, in a subset of individuals with multiple samples available at different time points before diagnosis, a longitudinal analysis was used. We assessed plasma TPS levels using enzyme-linked immunosorbent assay (ELISA) in 267 pre-diagnostic PDAC plasma samples taken up to 18.8 years before clinical PDAC diagnosis and in 320 matched healthy controls. TPS levels were also assessed in 25 samples at PDAC diagnosis. Circulating TPS levels were low both in pre-diagnostic samples of future PDAC patients and in healthy controls, whereas TPS levels at PDAC diagnosis were significantly increased (odds ratio 1.03; 95% confidence interval: 1.01–1.05) in a logistic regression model adjusted for age. In conclusion, TPS levels increase late in PDAC progression and hold no potential as a biomarker for early detection.

## 1. Introduction

Pancreatic ductal adenocarcinoma (PDAC) is an aggressive cancer type with a global overall 5-year survival of 9% [1]. A major reason for the poor prognosis is late-arising symptoms, and thus patients are diagnosed at advanced disease stages when curative surgery is no longer an option. However, the 5-year survival is also low (30–40%) for patients with localized disease where curative surgery is possible [2]. Sensitive biomarkers for detecting PDAC at an earlier stage are needed to improve the prognosis. Such biomarkers would lead to more patients being diagnosed at stages amenable for curative surgery.

The possibility of detecting PDAC early depends on the tumor progression rate. A mathematical model based on sequencing data from primary cancers and paired metastases suggested ≥10 years from initial mutation to founder cancer cell, ≥5 years for establishment of metastatic lesions, and another 2 years before the patients die [3]. In contrast, another study estimated that early- to late-stage PDAC progression takes only 1.3 years on average by comparing the mean age in relation to stage at diagnosis [4]. In addition, chromothripsis and polyploidization events, associated with aggressive and unstable tumors, seem to play a significant role in PDAC progression [5,6].

Carbohydrate antigen 19-9 (CA19-9) is a sialylated form of Lewis a antigen, and the ‘golden standard’ biomarker in PDAC diagnostics and clinical follow-up [7,8,9]. Potential PDAC biomarker candidates have been identified over the last 10 years; however, none have been introduced into clinical practice [10]. Different types of biomolecules, such as metabolomics [11,12,13,14] and microRNAs [15,16], have been analyzed in pre-diagnostic PDAC cohorts previously to identify early PDAC biomarker candidates or risk factors. Tissue polypeptide specific antigen (TPS) is a specific fragment of keratin 18, which belongs to type I intermediate filaments that are found in epithelia. TPS has previously been studied in epithelial-associated carcinomas, such as breast cancer [17,18,19,20], colorectal cancer [17,21,22], and PDAC [23,24,25,26,27,28,29,30]. We previously found circulating TPS levels to be higher in PDAC patients compared to healthy controls [25]. Furthermore, TPS levels decreased after surgery but remained significantly higher compared to healthy controls. In another study, TPS was elevated pre-operatively and suggested to hold greater potential than CA 19-9 for early diagnosis (stage I & II) of PDAC [24]. In contrast, others have found TPS to have a low ability to distinguish PDAC from benign and malignant hepatopancreatobiliary diseases [26].

In this study, the aim was to determine whether TPS holds potential as an early circulating biomarker of PDAC. We analyzed TPS levels in plasma samples from PDAC patients at diagnosis and in future patients up to 18.8 years prior to diagnosis. TPS levels were also analyzed in individual future PDAC patients over time. TPS levels were increased at PDAC diagnosis, but this increase was not observed in pre-diagnostic PDAC samples.

## 2. Materials and Methods

### 2.1. Ethics Statement

All participating subjects provided informed written consent. The ethical committee at Umeå University approved the study according to Helsinki Declaration of 1975.

### 2.2. Study Design

A cross-sectional case-control study in a pre-diagnostic cohort was designed to compare circulating TPS levels between healthy controls and future PDAC patients. For a subset of individuals, multiple samples were available, thus allowing for a longitudinal analysis of TPS levels over time. Circulating TPS levels were also assessed at time of PDAC diagnosis in plasma samples. This study followed the Strengthening the Reporting of Observational studies in Epidemiology (STROBE) guidelines [31].

### 2.3. Pre-Diagnostic Cohort

Pre-diagnostic PDAC plasma samples were obtained from the biobank of the Northern Sweden Health and Disease Study (NSHDS). The inclusion criterion was a histologically verified PDAC diagnosis with no previously reported malignancy. Each individual that developed PDAC by 31 December 2009 was matched to two healthy controls without malignant disease, based on sampling date (+/−1 year), age at diagnosis (+/−6 months), and sex (Table 1). The healthy controls were matched at the first sampling date of the future PDAC cases. PDAC diagnosis was defined as the date of first radiological finding of the tumor.

### 2.4. Diagnostic Cohort

Plasma samples were obtained at PDAC diagnosis (*n* = 26 samples from 22 individuals) from the Department of Surgery, Umeå University Hospital (Table 2). The plasma samples were collected before curative surgery or initiation of oncological treatment. Multiple samples were available for a subset of patients before treatment initiation but after diagnosis. Eight healthy control samples were collected from patients admitted to the department with non-malignant disease. Plasma TPS levels in these eight healthy controls have previously been analyzed [25]. Additional controls matched to the same individual were available among the controls of the pre-diagnostic cohort (*n* = 44), resulting in 52 healthy controls.

### 2.5. ELISA

We assessed TPS levels in plasma samples using TPS ELISA (IDL Biotech, Bromma, Sweden) according to the manufacturer’s protocol. All samples were run in duplicates. A coefficient of variation (%CV) limit of <15% between replicates was used, but for samples with TPS levels below the lowest reference value (<80 U/L), a higher %CV was accepted.

### 2.6. Statistical Analysis

Statistical analyses were performed in R Project for Statistical Computing (RRID:SCR_001905) version 4.0.3 [32]. Fisher’s exact test was used to compare sex distributions and Mann–Whitney to compare age between cases and controls in the diagnostic cohort. Since cases and controls were matched in the pre-diagnostic cohort, no statistical analyses were performed for comparing sex distribution or age. Since the time between sampling date and diagnosis differed between the PDAC cases in the pre-diagnostic cohort, we divided them into three smaller groups based on follow-up time to diagnosis (>10 years, 5–10 years, ≤5 years). Conditional logistic regression was performed for the pre-diagnostic cohort using the clogit function from the survival R package [33,34]. Logistic regression was performed for the diagnostic cohort using glm function. TPS and age were included in the models as independent variables, since age previously correlated with TPS levels [19]. Kendall’s method was used to correlate age and TPS in all healthy controls. The odds ratios for the regression models were obtained using R package epiDisplay [35]. One extreme outlier sample with a value of >6000 U/L was excluded among the cases in the diagnostic cohort. Kruskal–Wallis rank sum test was used for comparing circulating TPS levels between different PDAC stages at diagnosis. Spaghetti plot was generated using ggplot2 (RRID:SCR_014601) version 3.3.2 [36] and viridis version 0.5.1 packages [37,38] for TPS levels in plasma samples from individuals where samples were available both at PDAC diagnosis as well as before diagnosis (pre-diagnostic samples). A *p*-value < 0.05 was considered significant.

## 3. Results

We investigated the potential of circulating TPS levels as an early detection biomarker using a pre-diagnostic PDAC cohort with samples collected 1.6 months–18.8 years prior to diagnosis. Moreover, TPS levels were measured in plasma samples from patients at diagnosis and in healthy controls.

**Figure 1:**
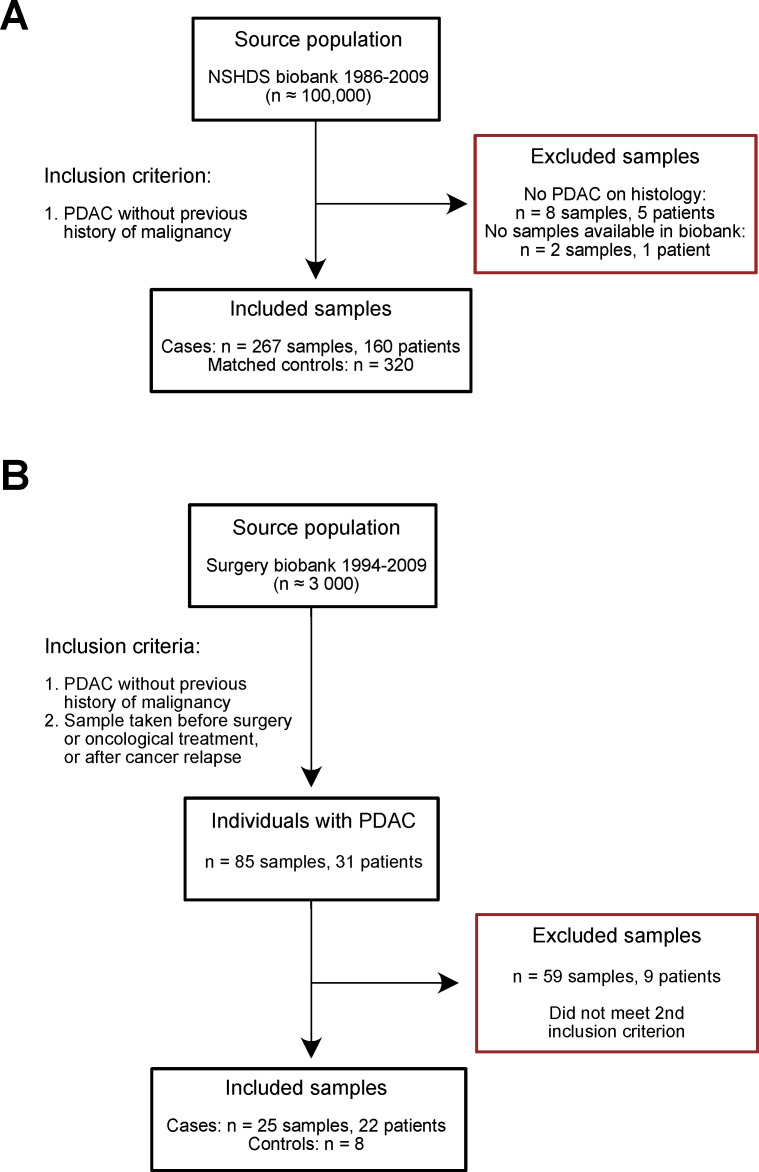
Flowchart of included and excluded samples in the (**A**) pre-diagnostic cohort and (**B**) diagnostic cohort. NSHDS = Northern Sweden Health and Disease Study.

Repeated samples were available for a subset of the future PDAC cases, and thus the total number of blood samples from cases were 267 (Figure 1A, *n* = 160 unique individuals). The healthy controls (*n* = 320) were matched to future cases at the first sampling point. Twenty-six PDAC samples from 22 patients, as well as 8 healthy controls, were retrieved from the Surgery biobank (Figure 1B).

**Figure 2:**
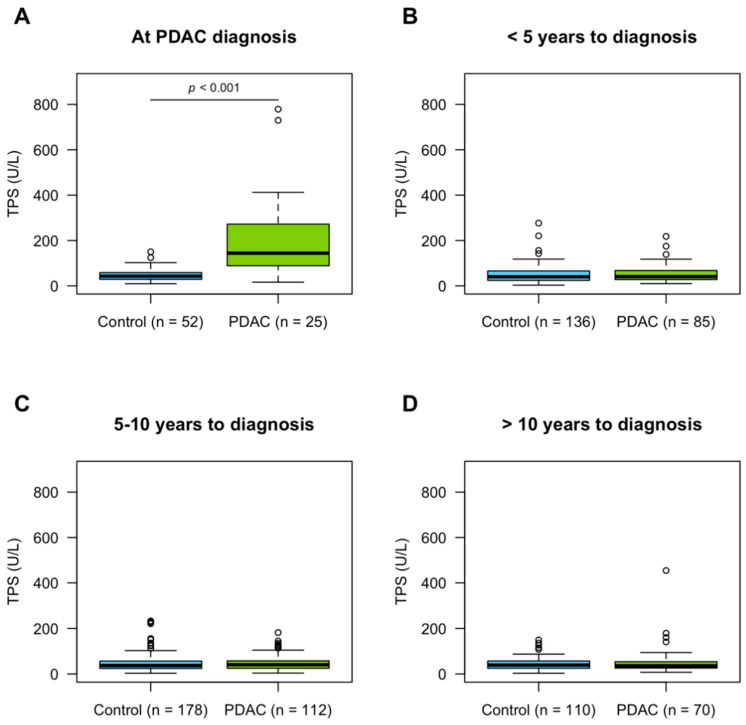
TPS levels in plasma samples stratified by time to PDAC diagnosis. The TPS levels were compared between cases and controls (**A**) at diagnosis (*p*-value from logistic regression model adjusted for age), (**B**) <5 years, (**C**) 5–10 years, and (**D**) >10 years prior to diagnosis.

**Figure 3:**
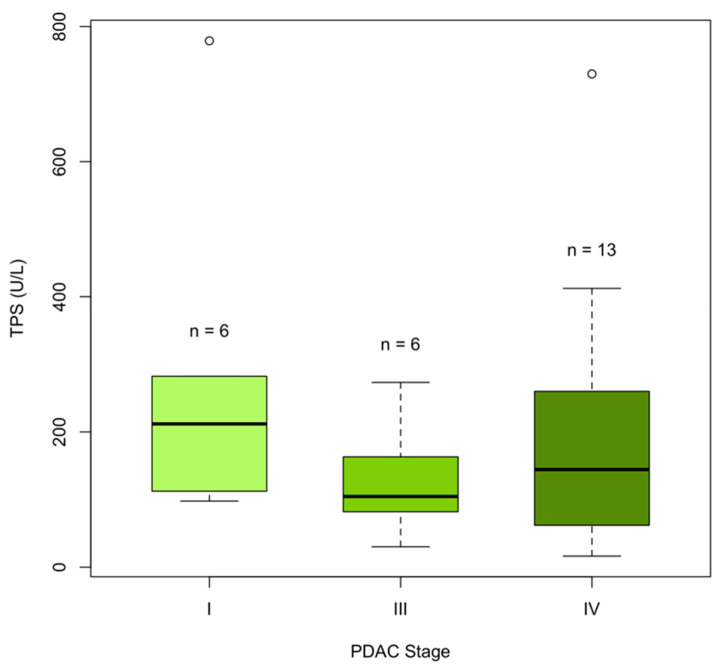
Circulating TPS levels according to PDAC stage in the diagnostic cohort (*n* = 25 cases). No difference in TPS levels was found between PDAC stages I, II, IV (Kruskal–Wallis rank sum test, *p*-value = 0.3). No PDAC samples were available at stage II.

As expected, circulating TPS levels were significantly elevated in PDAC patients at diagnosis (Table 3, Figure 2A, *p* < 0.001). The mean TPS levels were 208 ± 196 U/L in cases and 48 ± 28 U/L in controls. Age differed significantly between cases and controls in the diagnostic cohort (Table 2). Furthermore, age and TPS were significantly correlated in healthy controls (Figure S1), supporting previous findings [19] and highlighting the importance of adjusting for “age”. TPS levels were high at stage I, III, and IV PDAC (Figure 3).

TPS levels were low in pre-diagnostic PDAC samples and their matched controls when stratifying the cases into different time intervals from sample date to diagnosis (Table 3, Figure 2B–D). Further stratification of the pre-diagnostic samples according to time to diagnosis did not reveal any increase in TPS levels closer to the time point of diagnosis (Figure S2A). As PDAC patients are often diagnosed at late stages, we also stratified patients using time of death as endpoint. TPS levels in pre-diagnostic samples, however, remained low regardless of how cases were stratified. It should be noted though that no pre-diagnostic samples were taken within 6 months from death (Figure S2B).

**Figure 4:**
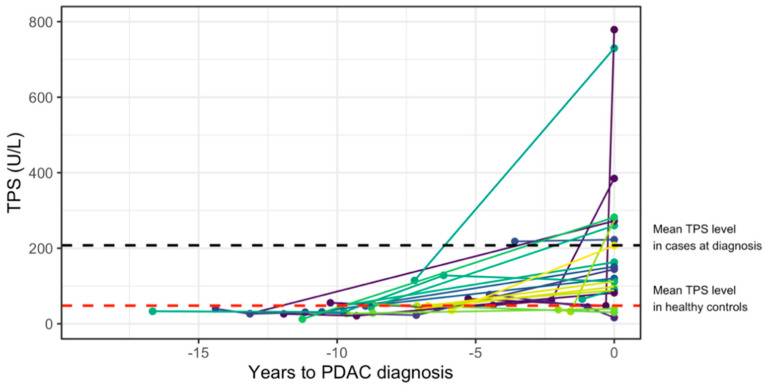
Longitudinal TPS plasma levels. Spaghetti plot of longitudinal plasma samples available at time of PDAC diagnosis and years before diagnosis in 22 individuals. The TPS levels are shown on the *y*-axis, and years until PDAC diagnosis are shown on the *x*-axis. Each line combines samples taken from the same individual. Time to diagnosis is presented as negative values for visual purposes.

TPS levels were also analyzed in longitudinal samples, that is, samples taken from the same individual prior to diagnosis and at diagnosis (Figure 4), in 22 individuals. A trend where circulating TPS levels increase at time of PDAC diagnosis was observed. Of note, one PDAC patient already had elevated TPS levels (>200 U/L) 3.6 years prior to PDAC diagnosis and survived for 57 days after diagnosis date. No further statistical analyses were conducted, however, due to the low number of samples and differences in sampling time points.

## 4. Discussion

PDAC is often discovered at an advanced stage, and only 20–30% of the patients undergo curative surgery. The 5-year survival also remains low in patients who have undergone surgery with curative intent, and this highlights the aggressive nature of this cancer type [2]. Early PDAC detection is crucial to improve the outcome of these patients. One approach is to identify potential biomarkers that can be used in screening settings to identify PDAC patients at an earlier stage in high-risk populations. In this study, TPS, a controversial biomarker in PDAC diagnostics, was evaluated for its potential as an early detection marker in a large pre-diagnostic PDAC cohort. We also assessed circulating TPS in a smaller PDAC cohort with samples obtained after clinical diagnosis, prior to surgery or oncological treatment, or after cancer relapse.

TPS levels were significantly increased at PDAC diagnosis, which has been shown previously [24,25]. No difference was found in plasma TPS levels at different PDAC stages (Figure 3), which is in agreement with Slesak et al. [24]. However, higher TPS levels in PDAC patients with lymph node or distant metastases was found by Talar-Wajnorowska et al. [29]. Increased TPS levels have also been shown in other metastasized primary cancers, such as breast cancer and colorectal cancer [17,18,39]. Since several studies have observed increased TPS levels in metastasized cancer, and we find high levels already at stage I PDAC, our results support the observation that PDAC is already a systemic disease at the early stages, and that even small tumors (<0.5 cm) can metastasize early [40].

The plasma TPS levels were low in the pre-diagnostic plasma samples. No indications of elevated TPS levels, even very close (6 months) to diagnosis, was found. The reason for these findings could be that the TPS increase occurs very late in PDAC progression, despite modelling studies showing a potentially long window for early detection from initiating mutations to death due to metastatic spread [3]. The present findings could be in line with TPS increases occurring during the fast PDAC progression where chromothripsis and large chromosomal rearrangements have already occurred [5]. This would explain why no evidence of increased TPS levels can be seen even close to the date of diagnosis.

One limitation of this study is the sample sizes of the longitudinal cohort and diagnostic cohort; however, the primary focus was to investigate TPS in the large pre-diagnostic cross-sectional cohort.

## 5. Conclusions

Circulating TPS levels do not hold promise as a potential biomarker for early PDAC detection in a large cohort of pre-diagnostic plasma samples from future PDAC patients.

## Figures and Tables

**Table 1:** Clinical characteristics of pre-diagnostic cohort.

Variable	Cases (*n* = 160)	Controls (*n* = 320)
Mean age at blood sample collection (years)	55	55
Sex		
Men, *n*	57	114
Women, *n*	103	206
TNM stage at diagnosis (7th ed of AJCC staging)		
Stage I	12	NA
Stage II	11	NA
Stage III	37	NA
Stage IV	100	NA
Grade at diagnosis		
Low	33	NA
Intermediate	44	NA
High	4	NA
Information missing	79	NA
Surgical treatment		
None	111	NA
Curative	23	NA
Palliative	25	NA
Information missing	1	NA

NA = not applicable.

**Table 2:** Clinical characteristics of diagnostic cohort.

Variable	Cases (*n* = 22)	Controls (*n* = 52)
Mean age at blood sample collection (years) *	61	52
Sex ^#^		
Men, *n*	9	21
Women, *n*	13	31
TNM stage (7th ed of AJCC staging)		
Stage I	6	NA
Stage II	0	NA
Stage III	6	NA
Stage IV	10	NA
Surgical treatment		
None	11	NA
Curative	7	NA
Palliative	4	NA

* Mann–Whitney test, *p* < 0.001, not analyzed in the pre-diagnostic cohort since cases and controls were matched. ^#^ Fisher’s exact test, *p* = 1, not analyzed in the pre-diagnostic cohort since cases and controls were matched. NA = not applicable.

**Table 3:** Estimates and odds ratios for TPS adjusted for age.

Stratum (Number of Samples)	Crude Estimate ^a^	Crude OR (95% CI)	Adjusted Estimate ^a^	Adjusted OR (95% CI)
At diagnosis (25 PDAC, 52 controls)	0.033 (*p* < 0.001)	1.03 (1.02–1.05)	0.030 (*p* < 0.001)	1.03 (1.01–1.05)
<5 years to diagnosis (85 PDAC, 136 controls)	0.002	1.00 (0.99–1.01)	0.006	1.01 (1.00–1.02)
5–10 years to diagnosis (112 PDAC, 178 controls)	0.0003	1.00 (0.99–1.01)	−0.004	1.00 (0.99–1.00)
>10 years to diagnosis (70 PDAC, 110 controls)	0.004	1.00 (1.00–1.01)	0.005	1.00 (1.00–1.01)

PDAC = pancreatic ductal adenocarcinoma, OR = odds ratio, CI = confidence interval. ^a^ The estimate corresponds to fitted β-coefficients in the regression models.

## Data Availability

The data presented in this study are available on request from the corresponding author. The data are not publicly available due to the Swedish regulations regarding handling of personal health information.

## Supplementary Materials

The following are available online at https://www.mdpi.com/article/10.3390/cancers13215321/s1, Figure S1: Kendall’s correlation between age and TPS levels in healthy controls (*n* = 328), Figure S2: Circulating TPS levels stratified into groups according to (A) follow-up time from sample date to PDAC diagnosis, or (B) follow-up time from sample date to time of death, along with TPS levels at diagnosis.
