# Supplementary material for: Circulating Tissue Polypeptide-Specific Antigen in Pre-Diagnostic Pancreatic Cancer Samples
Source: Cancers (Basel). 2021 Oct 23;13(21):5321. doi: 10.3390/cancers13215321 (PMC8582400; doi:10.3390/cancers13215321)

Supplementary Information

Figure S1. Kendall's correlation between age and TPS levels in healthy controls (n = 328).

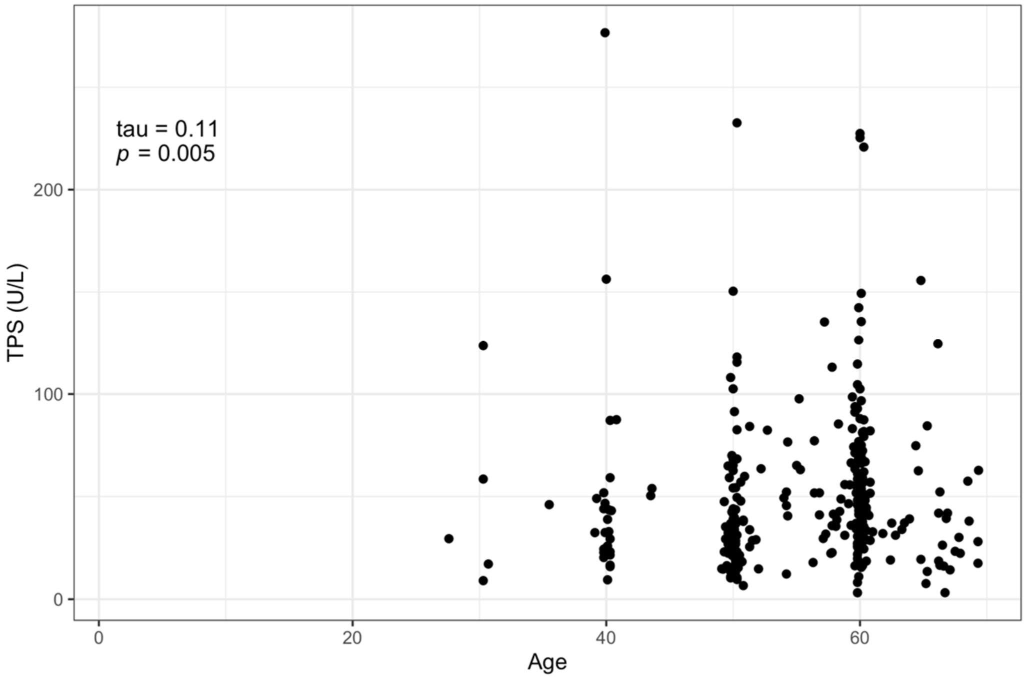

Figure S2. Circulating TPS levels stratified into groups according to (A) follow-up time from sample date to PDAC diagnosis, or (B) follow-up time from sample date to time of death, along with TPS levels at diagnosis. The healthy controls (Ctrl) include only the pre-diagnostic controls. y = years, m = months.

A

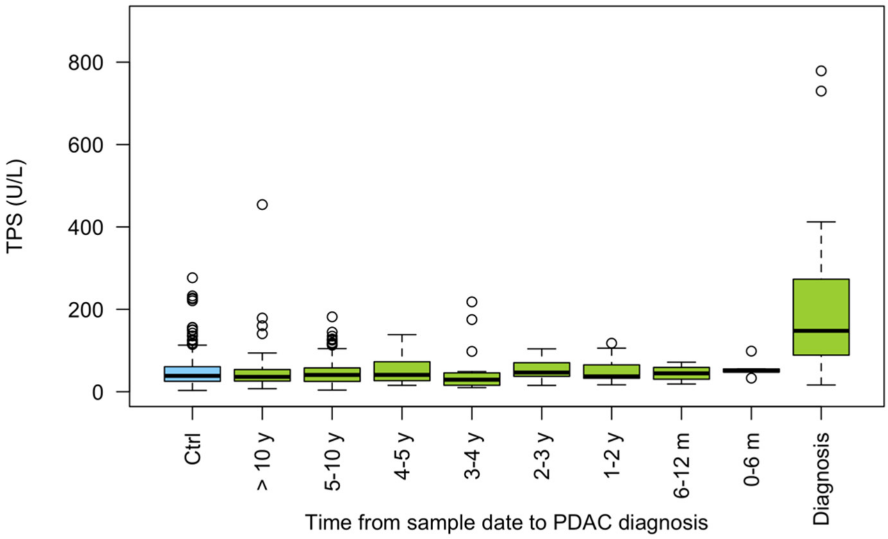

B

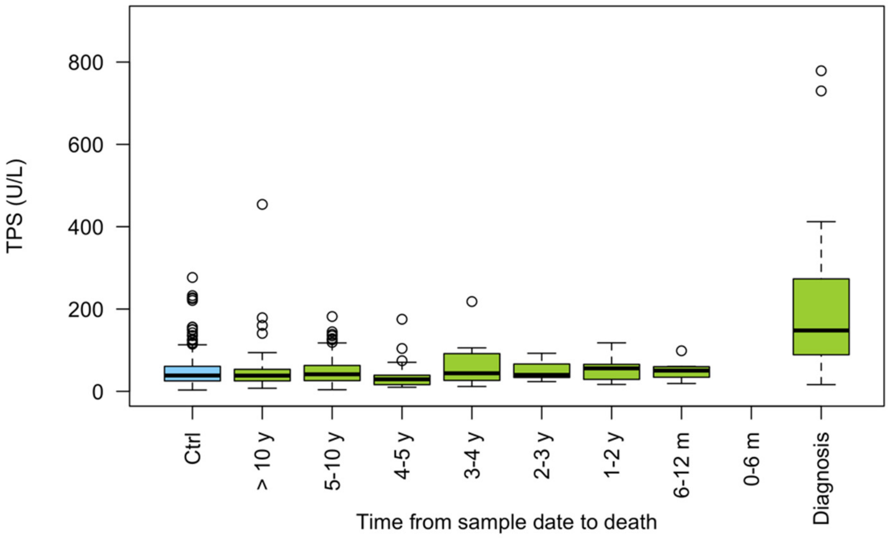

Supplement: Supplementary file 1 [file cancers-13-05321-s001.zip › cancers-1385070-supplementary.pdf]
